# Development of the STAR Evaluation System for Assessing Bicycle Helmet Protective Performance

**DOI:** 10.1007/s10439-019-02330-0

**Published:** 2019-08-01

**Authors:** Megan L. Bland, Craig McNally, David S. Zuby, Becky C. Mueller, Steven Rowson

**Affiliations:** 1 Department of Biomedical Engineering and Mechanics, Virginia Tech, Center for Injury Biomechanics, 440 Kelly Hall, 325 Stanger Street, Blacksburg, VA 24061 USA; 2 Insurance Institute for Highway Safety, 988 Dairy Road, Ruckersville, VA 22968 USA

**Keywords:** Concussion, Cycling, Injury risk, Impact, Biomechanics

## Abstract

Cycling is a leading cause of mild traumatic brain injury in the US. While bicycle helmets help protect cyclists who crash, limited biomechanical data exist differentiating helmet protective capabilities. This paper describes the development of a bicycle helmet evaluation scheme based in real-world cyclist accidents and brain injury mechanisms. Thirty helmet models were subjected to oblique impacts at six helmet locations and two impact velocities. The summation of tests for the analysis of risk (STAR) equation, which condenses helmet performance from a range of tests into a single value, was used to summarize measured linear and rotational head kinematics in the context of concussion risk. STAR values varied between helmets (10.9–25.3), with lower values representing superior protection. Road helmets produced lower STAR values than urban helmets. Helmets with slip planes produced lower STAR values than helmets without. This bicycle helmet evaluation protocol can educate consumers on the relative impact performance of various helmets and stimulate safer helmet design.

## Introduction

Concussion, a mild traumatic brain injury (mTBI), has gained a national and international spotlight as long-term detrimental sequelae of repeated mTBI are brought to the public’s eye.^30^ Studies estimate that up to 3.8 million sport-related concussions occur each year in the United States.^10^ While most of the research and public awareness surrounding concussive injury in sports focuses on American football, injury surveillance systems indicate that cycling is among the leading causes of sport- or recreation-related concussions treated in the US.^8^ Cycling has increased in popularity due to its many health and environmental benefits, with approximately 103.7 million Americans ages three and older having ridden a bicycle in 2015.^15^ Its increasing popularity is paralleled by increasing injury rates, accounting for an estimated $24.4 billion in US healthcare costs in 2013.^15^,^16^ Fortunately, helmet use has been demonstrated to reduce risk of head injury for cyclists involved in a crash.^22^,^29^

Present head injury safety standards aim to reduce risk of catastrophic injury or death by placing a limit on headform linear acceleration-related metrics during helmet impact testing. The Consumer Product Safety Commission (CPSC) standard is mandatory for bicycle helmets in the US,^9^ and dictates that helmets must limit peak linear acceleration (PLA) to less than 300 g—a level associated with > 50% risk of skull fracture or severe brain injury.^23^ Linear acceleration-derived metrics are generally based on early human cadaver and animal testing. As brain injury was often observed in instances of skull fracture, it was deduced that brain injury could also be predicted by linear acceleration.^17^ However, research has since suggested that the mechanisms of mTBI are more complex than can be described by linear acceleration alone, and that head rotation plays a large role in producing injury.^19^ Linear acceleration has been correlated to the development of transient intracranial pressure gradients resulting in focal injury, while rotational kinematics have been correlated to shearing of the brain tissue resulting in diffuse injury.^19^,^20^ Real-world head impacts involve both linear and rotational components, and metrics that include both are good predictors of concussion.^35^,^44^ As concussions are common in cyclist crashes,^8^ there is value in assessing a helmet’s ability to reduce risk of concussion in addition to the severe injury levels addressed by standards.

The CPSC standard and other nations’ safety standards measure linear acceleration during drop tests using a linear accelerometer at the center of gravity (CG) of a metal headform.^9^,^13^ The CPSC test setup is constrained to linear motion and does not reflect the nature of head impact rotations that a cyclist might experience. A cyclist’s head nearly always approaches a surface at an angle during a crash, termed an oblique impact.^5^,^41^ Standards specify drop tests in a direction normal to the impact surface. Oblique impact testing using a biofidelic headform capable of measuring both linear and rotational kinematics would enhance assessment of helmet performance in reducing concussion risk. Several oblique rigs have already been developed for bicycle helmet testing.^1^,^26^,^27^ Establishing an oblique impact protocol for bicycle helmets could help manufacturers design around these common conditions as well as standards-prescribed conditions.

Despite standards addressing risk of catastrophic injury and recent efforts to assess helmet efficacy in reducing risk of milder injuries, limited comparative data are available to consumers indicating which bicycle helmets afford better protection. The existing objective impact data comparing helmet models are either limited to standards testing or only evaluate a relatively small subset of helmets on the US market.^1^,^3^,^26^,^40^ While most helmets are designed with similar materials, typically including a polycarbonate shell and expanded polystyrene (EPS) liner that permanently crushes to absorb energy upon impact, the many possible styles of bicycle helmets produce wide ranging design features, and previous research has demonstrated considerable differences in the ability of commercially-available helmets to reduce head injury risk.^1^,^3^,^26^,^40^ To help reduce incidence of concussion in cycling and to stimulate improved helmet design for both mild and severe injury, consumers should have access to biomechanical data differentiating helmet performance that is informed by real-world cyclist impact conditions and injury mechanisms.

The Summation of Tests for the Analysis of Risk (STAR) assessment method is a biomechanically-based helmet evaluation protocol that quantifies the ability of individual helmet models to reduce incidence of concussion. This evaluation scheme has previously been employed for football and hockey helmets^32^,^34^ and operates on two fundamental principles. First, a battery of laboratory tests informed by field-driven impact data are conducted, and test results are weighted based on how frequently the given impact may occur on the field. Second, measured linear and rotational kinematics are related to injury risk such that helmets that more effectively reduce kinematics produce lower concussion risks. These principles are supported by a retrospective study that found lower on-field concussion rates associated with a football helmet that better-reduced laboratory head impact kinematics in comparison to a helmet that produced higher kinematics.^37^ The purposes of the present study are to describe the development of a similar STAR evaluation scheme for bicycle helmets, to explore its application using a large array of bicycle helmets on the US market, and to assess how helmet design influences impact performance.

## Materials and Methods

### Impact Testing

**Figure 1:**
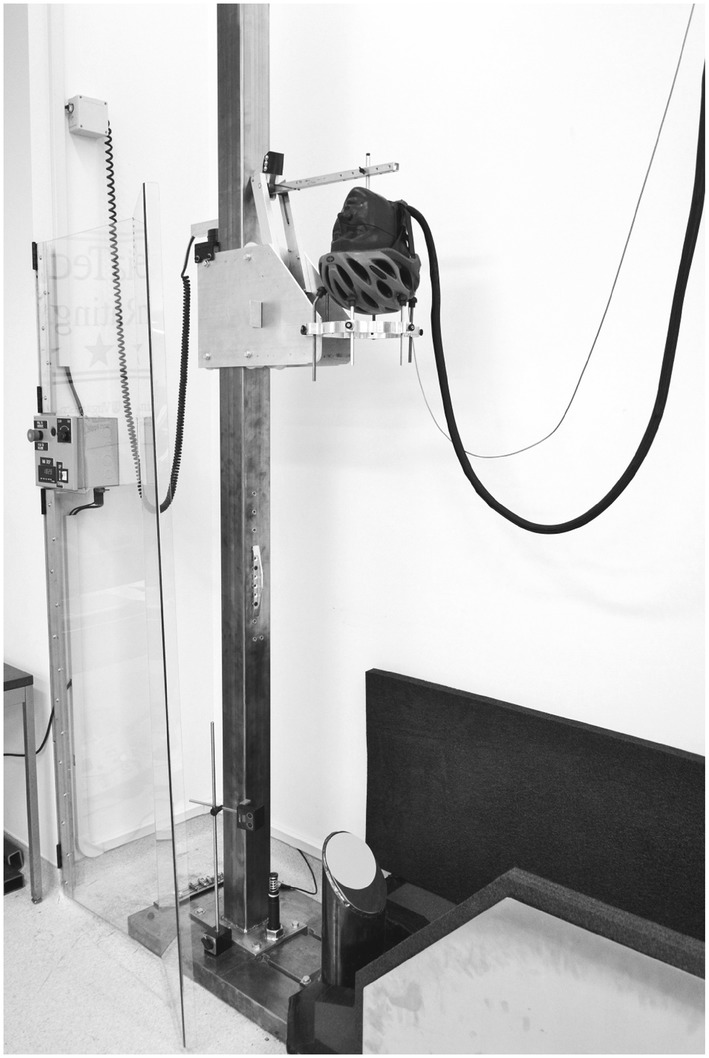
Custom impact rig used for STAR testing. A helmeted headform is dropped onto an angled anvil to generate an oblique impact.

A custom oblique drop tower was developed for STAR testing (Fig. 1), wherein a helmeted headform was dropped onto a 45° steel anvil to generate equal normal and tangential incident velocities. This angle falls central to a range of reported cyclist head impact angles from simulation studies,^5^,^31^,^41^ and evenly challenges a helmet’s ability to mitigate normal and tangential forces. Sandpaper was adhered to the anvil surface to simulate road friction (80 grit^11^), and was replaced after every fourth test. Prior to each test, a medium National Operating Committee on Standards for Athletic Equipment (NOCSAE) headform was fitted with a helmet so that the rim (lower front edge) was 2.5 cm above the brow line. The dial fit was adjusted, if applicable, until slight resistance was met, then retention straps were tightened until nearly taut under the headform. The centeredness of the helmet was checked using headform anatomical planes. The helmeted headform was then positioned on a support ring constrained to the drop tower and secured in place from above using an additional support arm, which mechanically released just prior to impact. The support ring passed around the outside of the anvil upon impact.

No anthropomorphic test device (ATD) neck or effective torso mass was used, similar to a number of previous cyclist oblique head impact studies.^2^,^26^,^27^,^40^ Although some studies have used a Hybrid III (HIII) neck for this type of testing,^1^,^22^ oblique impacts may subject the neck to considerable axial compression for certain impact locations, a scenario in which the HIII neck is known to be overly stiff.^2^,^28^,^38^ It has been further suggested that human necks have minimal effect on initial head impact response during head-first impacts due to the high loads and short durations involved.^12^,^28^,^38^

**Figure 2:**
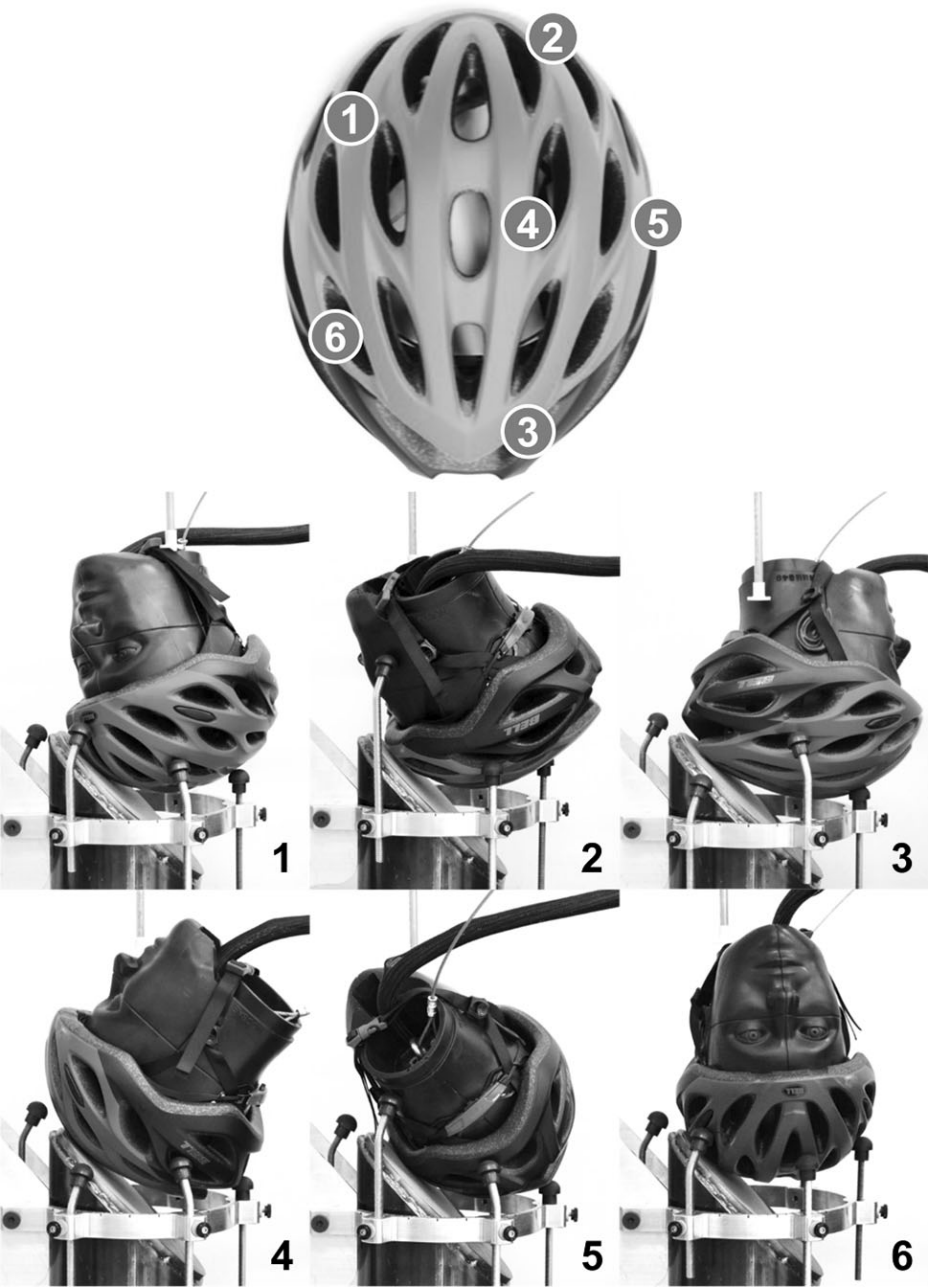
The six impact locations selected for testing (top), along with required orientations of the helmeted headform in the support ring to impact each location (bottom). Locations 1, 2, and 6 represent body-driven impacts, in which the head leads the body, locations 3 and 5 represent skidding-type impacts, and location 4 represents an impact from flipping over the handlebars.

Impact conditions were selected to reflect those common in cyclist crashes as determined by helmet damage replication studies and computational simulation studies.^5^,^31^,^39^,^41^,^42^ Six locations dispersed around the helmet were selected to assess helmet performance over a range of impact scenarios (Fig. 2). Locations 2 and 5 fall at the helmet rim, a commonly impacted area that is not considered in standards testing.^5^,^31^,^39^,^41^,^42^ Four locations were set to the front and sides of the helmet, which are very commonly impacted in cyclist crashes.^5^,^41^ Locations were set > 120 mm apart, which the CPSC suggests is sufficient distance to prevent overlap of damage profiles from previous tests.^9^ To ensure precision in impacting each location, a dual-axis inclinometer (DMI600, Omni Instruments, Dundee, UK) was mounted parallel to the base of the headform during positioning to mark headform orientation (Table 1). The support ring was inscribed with 5° increments to specify Z rotation as well.

**Table 1:** Orientation specifications of the NOCSAE headform to impact each desired location.

Location	*X* (deg)	*Y* (deg)	*Z* (deg)
1	17.2	1.7	− 75
2	− 31.4	− 13.3	60
3	− 22.5	− 2.9	− 170
4	− 7.0	43.7	15
5	− 44.0	31.9	180
6	2.6	12.2	− 110

*X* and *Y* specifications are determined using a dual-axis inclinometer, while the Z specification is determined by extending the sagittal line on the face of the NOCSAE until it intersects the support ring, which is inscribed in 5° increments (headform facing drop tower is 0°, clockwise is positive)

Impact velocities were assigned based on helmet damage replication study data.^39^,^42^ These studies gathered helmets from manufacturer warranty replacement programs or hospitalized cyclists, recreated the liner crush depth using standards test equipment, and recorded associated normal velocities. As liner crush depth is well-correlated with normal velocity,^25^ oblique impacts with the same normal velocities can be assumed to generate similar crush depths (using similar masses). The 50th and 90th percentile replication study normal velocities (3.4 and 5.3 m/s, respectively) were thus selected for the present study in order to simulate average and severe impact energies. On the 45° anvil, these were associated with resultant velocities of 4.8 and 7.4 m/s and tangential velocities of 3.4 and 5.3 m/s. The tangential velocities represent ≃ 25th and 50th percentiles of cyclist accident simulation data.^5^,^31^,^41^ Simulations likely represent more severe impacts given that they rely on detailed accident reports, which are often only collected for more severe accidents. Two trials of the 12 location-velocity impact configurations were conducted for every helmet model, with four samples per helmet model so that each sample was impacted only once per location. Each sample was tested in order of location (location 1 tested first, 6 last), with three low-velocity and three high-velocity impacts per sample.

Linear and rotational kinematics were recorded at 20 kHz during each test using three linear accelerometers (Endevco 7264B-2000, Meggitt Sensing Systems, Irvine, CA) and a tri-axis angular rate sensor (ARS3 PRO-18K, DTS, Seal Beach, CA) at the CG. Linear acceleration was filtered according to SAE J211 using a channel frequency class (CFC) of 1000, while rotational velocity was filtered at a CFC of 175. The latter CFC has been shown to optimize rotational responses relative to the more conventional nine accelerometer array (9AA).^2^,^7^ An ARS was selected over a 9AA because it is more compatible with the NOCSAE headform’s small instrumentation channel. Resultant PLA and peak rotational velocity (PRV) were then averaged across the two trials at each impact configuration and used to estimate risk of concussion, which was then implemented into the STAR equation.

### Bicycle STAR Equation

**Figure 3:**
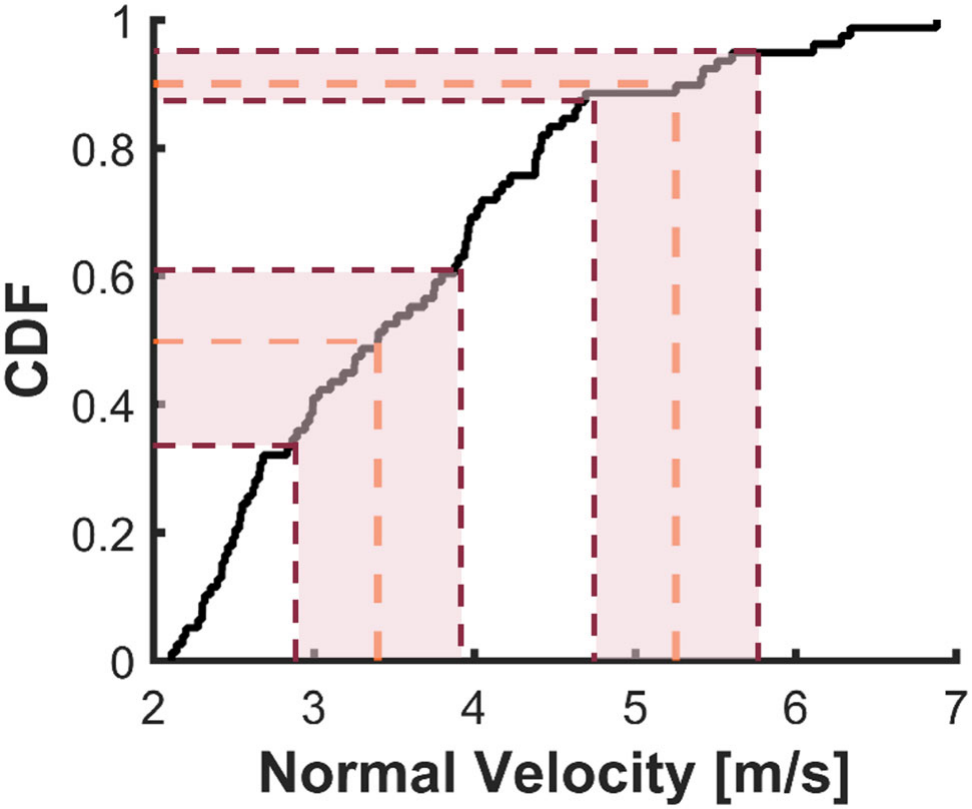
CDF of digitized data from helmet damage replication studies. STAR test velocities were chosen based on the 50th and 90th percentiles (3.4 and 5.2 m/s normal velocities, respectively). A 0.5 m/s range from either velocity was then mapped to the CDF (shaded region), and the number of impacts occurring within that range was divided evenly by the six impact locations to determine weighting factors for the STAR equation. The 3.4 ± 0.5 m/s range encompassed 38.0 impacts, so all low speed impacts were weighted by 6.33. The 5.2 ± 0.5 m/s range encompassed 9.4 impacts, so all high speed impacts were weighted by 1.57.

The STAR equation summarizes helmet performance from a range of tests into a single value. Bicycle STAR follows the guidelines of the previously-published football and hockey STAR equations,^32^,^34^ with slight modifications Eq. (1). An exposure term, *E*, weights each impact configuration based on its frequency in real-world impact scenarios. Each configuration is comprised of a location, *L*, and velocity, *V*. Locations were equally weighted to ensure helmets are not under-designed in any one location, while assigning four of the six locations to the front and sides (two of these at the rim) ensures that helmets are designed robustly in these commonly-impacted locations. Impact velocities were weighted based on the number of impacts within ± 0.5 m/s of the target normal velocities based on a cumulative distribution function (CDF) of damage replication data (Fig. 3). Per 100 total head impacts, 38.0 impacts fell within 3.4 ± 0.5 m/s and 9.4 impacts within 5.2 ± 0.5 m/s. The number of impacts were split evenly among the 6 locations to yield final weightings of 6.33 for each low-speed impact and 1.57 for each high-speed impact.

1 $$Bicycle STAR = \mathop \sum \limits_{L = 1}^{6} \mathop \sum \limits_{V = 1}^{2} E\left( {L,V} \right)*R\left( {a,\omega } \right)$$

The other major term in the STAR equation is concussion injury risk, *R*, which is a function of PLA, *a*, and PRV, *ω*. The PLA- and peak rotational acceleration (PRA)-based risk function used in hockey STAR was generated using logistic regression of head impact data from high school and collegiate football players, and accounts for underreporting of concussion.^35^ For bicycle STAR, this function was modified using a previously-published linear relationship between PRV and PRA based on six degree-of-freedom sensor data in football head impacts.^36^ PRV is an attractive rotational metric as it involves less inherent measurement variability than PRA, allows for duration of loading to be accounted for, and has recently been shown to be the best correlate to strain development in the brain leading to concussion.^18^,^20^ Equation (2) shows the updated risk function with the PRA-PRV transformation carried through.

2 $$R\left( {a,\omega } \right) = 1/(1 + e^{ \wedge }\left( { - \left( { - 10.2 + 0.0433a + 0.19686\omega - 0.0002075a\omega } \right)} \right)$$

Per impact configuration, risk was calculated using the average PLA and PRV and then multiplied by the respective exposure weighting. Weighted risks were summed to yield a STAR value for each helmet model Eq. (1). Each helmet’s STAR value estimates an incidence of concussion from the given array of real-world impact conditions.

### Helmet Models

Thirty popular CPSC-certified bicycle helmet models were purchased for STAR testing (Table 2), with sizes selected based on the NOCSAE head circumference. Fourteen brands were represented. Manufacturer suggested retail price (MSRP) at the time of purchase ranged from $10–250, and styles were dichotomized into road and urban categories. Road helmets contain an elongated, aerodynamic shape, substantial venting, and thin, flexible shells, while urban helmets are characterized by a more rounded shape with less venting and thicker, stiffer shells. Several of the road helmets were advertised as multi-sport or mountain bike helmets; these were placed in the road category for their substantial venting and thinner shells, which made them more similar to road helmets from a design standpoint. Many of the helmets contained Multi-directional Impact Protection System (MIPS) technology, a patented helmet insert designed to create a slip-plane layer between the wearer’s head and the rest of the helmet during impact. The intended purpose of the slip plane is to reduce rotational forces experienced by the head and thereby the resulting brain injury risk. Visors and other extraneous attachments were removed prior to testing, and internal helmet features (i.e., retention system, MIPS) were re-secured between tests when necessary.

**Table 2:** Helmet models selected for STAR testing.

Make	Model	MSRP [$]	Style	MIPS
Bell	Division	40	Urban	No
Bell	Draft MIPS	60	Road	Yes
Bell	Reflex	10	Road	No
Bell	Stratus MIPS	150	Road	Yes
Bern	Brentwood	70	Urban	No
Bern	Watts	60	Urban	No
Bontrager	Ballista MIPS	200	Road	Yes
Bontrager	Quantum MIPS	100	Road	Yes
Bontrager	Solstice	40	Road	No
Electra	Electra Helmet	70	Urban	No
Garneau	Le Tour II	50	Road	No
Garneau	Raid MIPS	120	Road	Yes
Giro	Foray MIPS	85	Road	Yes
Giro	Revel	45	Road	No
Giro	Savant	100	Road	No
Giro	Sutton MIPS	100	Urban	Yes
Giro	Synthe	250	Road	No
Kali	City	125	Urban	No
Lazer	Genesis	100	Road	No
Nutcase	Street	70	Urban	No
POC	Octal	200	Road	No
Schwinn	Flash	30	Road	No
Schwinn	Thrasher	14	Road	No
Scott	ARX Plus	100	Road	Yes
Smith	Overtake	163	Road	No
Specialized	Centro	60	Road	No
Specialized	Chamonix MIPS	75	Road	Yes
Specialized	Evade II	250	Road	No
Specialized	Prevail II	225	Road	No
Triple 8	Dual Certified MIPS	75	Urban	Yes

MSRP reflects the price provided by manufacturer websites at the time of purchase

### Statistical Analysis

Kinematic variance across trials one and two was assessed per impact configuration for each helmet as the range in PLA or PRV divided by the mean. Ten additional third trials were conducted spanning a range of impact configurations and helmets. While these trials decreased standard error by increasing the sample size, they had minimal effects on average PLA and PRV (≤ 2.9 g and ≤ 0.6 rad/s on average, respectively). It was thus deemed that two trials provided sufficient data for the present analysis while minimizing practicality burdens. The influence of impact location and velocity on kinematics and injury risks were assessed using ANOVA (Tukey’s HSD *post hoc*). Comparisons of STAR values based on helmet style and MIPS were assessed using unbalanced ANOVA (type III SS), while trends between STAR and helmet price were investigated using correlation analysis.

Variance in a helmet’s STAR value was investigated by propagating the kinematic variance through the STAR calculation. In calculating a STAR value, risk is computed using average PLA and PRV across the two trials for each configuration. To assess variance, two risks were instead calculated per configuration using the PLA and PRV results specific to each trial. Alternative STAR values were then calculated using only one of the two risk values for each configuration. Every permutation of first or second risk values for all 12 configurations was evaluated to yield possible STAR outcomes (2^12^ = 4096 permutations per helmet). Variance in STAR was assessed using the 95th percentile confidence intervals (95% CI) of these permutations.

## Results

**Figure 4:**
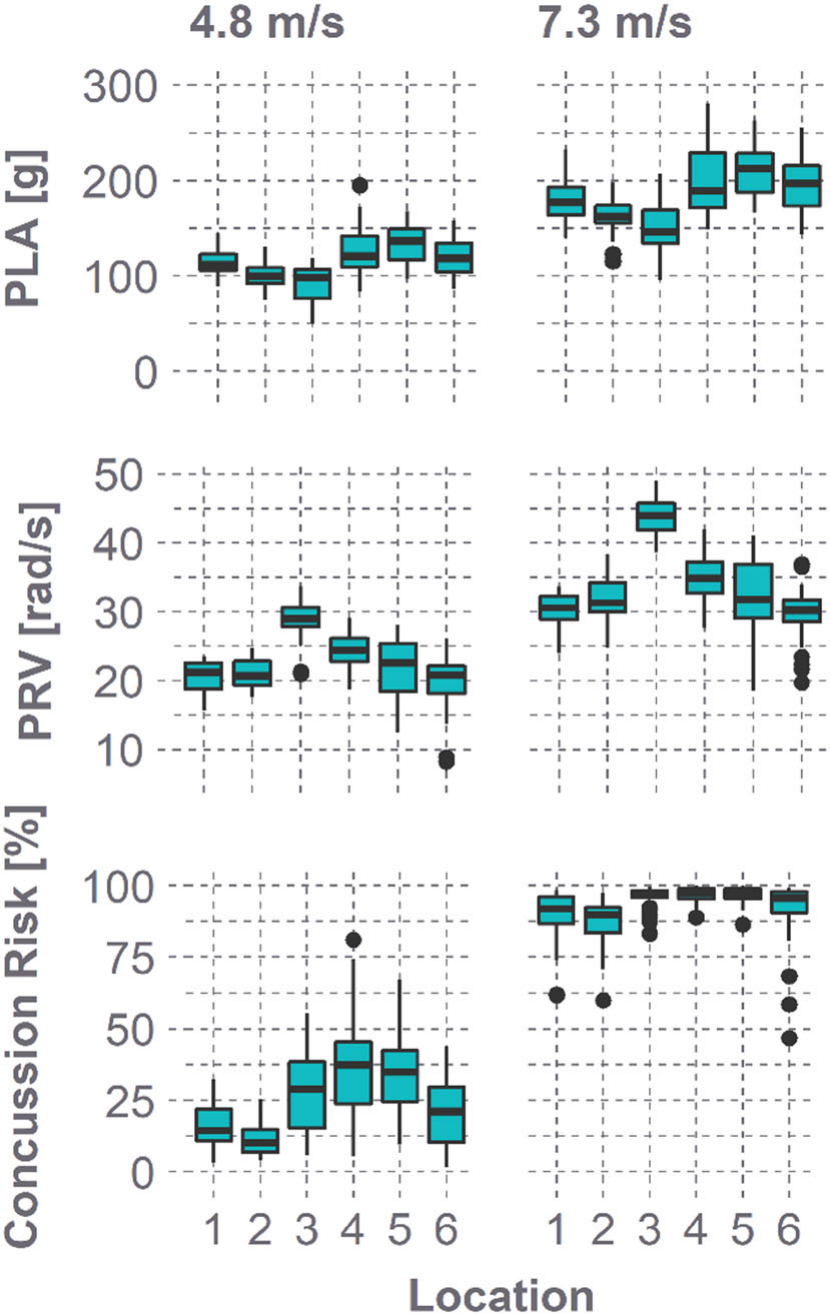
PLA, PRV, and concussion risk distributions for each impact configuration. Concussion risk was computed using a bivariate function including both PLA and PRV.

The 30 helmets evaluated produced wide ranges in kinematics across all configurations (Fig. 4). PLA averaged 114.0 ± 22.8 g at 4.8 m/s and 183.4 ± 33.5 g at 7.3 m/s, while PRV averaged 22.8 ± 4.2 rad/s at 4.8 m/s and 33.9 ± 5.9 rad/s at 7.3 m/s. Location 3 was associated with significantly lower PLAs and higher PRVs than most other locations (*p* < 0.05). Variance in PLA between trials one and two averaged 4.6 ± 4.1%, while PRV variance averaged 3.9 ± 4.1%. Variance was not significantly greater at either velocity (*t* test, *p *> 0.18), nor was PLA variance greater at later locations (correlation test, *R* = 0.03, *p* = 0.55). PRV variance increased significantly with later locations, although the correlation was not strong (*R *= 0.26, *p *< 0.01). Impact duration was comparable across velocities, averaging 9.7 ± 1.3 and 9.4 ± 1.7 ms at 4.8 and 7.3 m/s, respectively. Kinematics also varied considerably within single configurations, producing wide ranges in concussion risks that spanned up to 76% risk (location 4, 4.8 m/s). Risk averaged 24.9 ± 15.8% at 4.8 m/s and 92.8 ± 8.2% at 7.3 m/s. Location 2 produced lower risk values than most other impact locations (*p* < 0.05).

**Figure 5:**
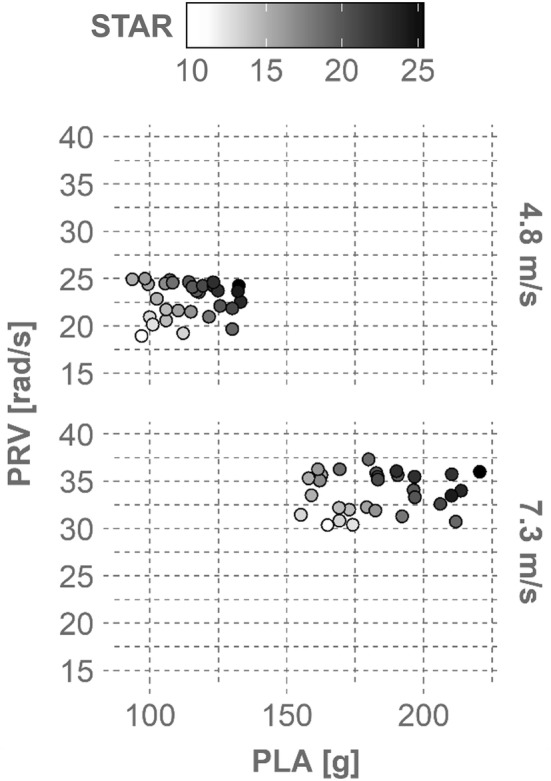
Effect of kinematic results on STAR values. Shown are PLA and PRV results averaged across impact locations for every helmet. Each helmet is represented by one circle per velocity, with its shade determined by its overall STAR value (e.g., the Ballista MIPS produced the lowest STAR value of 10.9 and is represented by a white circle near the lower left corner of each plot). STAR values increase with increasing kinematics.

**Figure 6:**
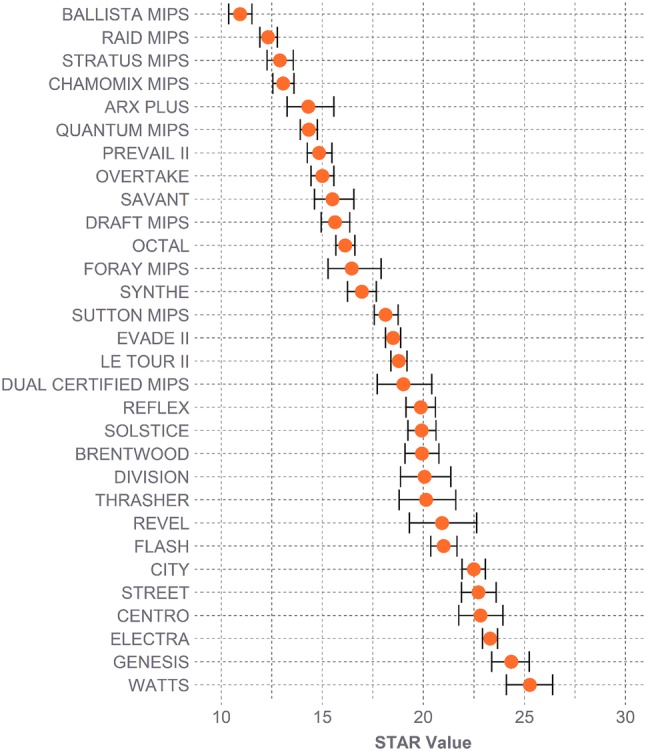
Range in STAR values across all helmet models. Lower STAR values indicate reduced incidence of concussion, and thereby enhanced protection. Error bars represent 95% CI ranges.

Helmets that better reduced kinematics generated lower risks, which in turn produced lower STAR values (Fig. 5). STAR ranged from 10.9 (Ballista MIPS, 95% CI: [10.4, 11.5]) to 25.3 (Watts, 95% CI: [24.1, 26.4]) (Fig. 6). Across all helmets, both velocities contributed evenly to the STAR values; weighted risks from 4.8 m/s impacts accounted for 50.3 ± 8.9% of STAR values, and weighted risks from 7.3 m/s impacts accounted for 49.7 ± 8.9%. Locations contributed relatively evenly to STAR as well, with the average contribution ranging from 11.6 ± 2.4% at location 2 to 21.2 ± 3.2% at location 4.

**Figure 7:**
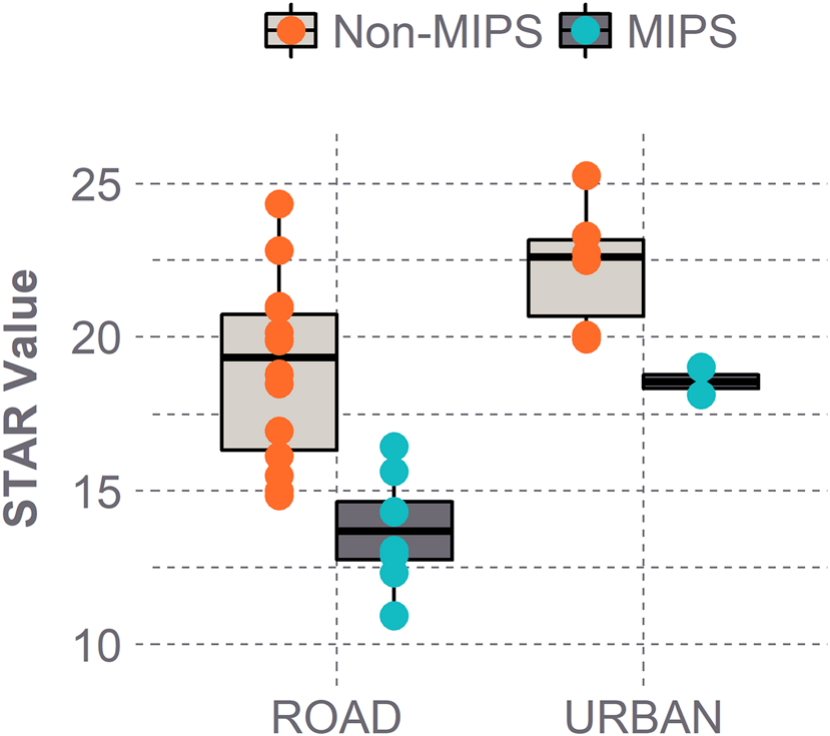
STAR value distributions based on style and MIPS. Road helmets with MIPS produced the lowest STAR values and thereby offer the greatest protection. MIPS helmets generated lower STAR values than non-MIPS helmets for both styles.

Of the 14 brands represented in this set of helmets, no single brand dominated the higher or lower STAR values. Urban helmets generally produced greater STAR values than road helmets (*p *< 0.01), with average STAR values of 21.4 ± 2.4 and 17.0 ± 3.6, respectively (Fig. 7). MIPS helmets produced lower STAR values than non-MIPS helmets (*p *< 0.01), averaging 14.7 ± 2.6 vs. 19.9 ± 3.1. There was no significant interaction between helmet style and MIPS (*p* = 0.53). Road helmets with MIPS generated the lowest STAR values overall (13.7 ± 1.8). Additionally, STAR values showed a slight negative correlation with MSRP, suggesting that more expensive helmets were associated with slightly greater protection. Although this trend was significant (*p *= 0.01), considerable variance was observed (*R*^2 ^= 0.22), with several lower-priced helmets receiving low STAR values and *vice versa*.

## Discussion

The present study details an objective evaluation protocol for bicycle helmets and examines its application using 30 popular helmets in the US. Impacts at the two velocities and six locations generated a wide range in head kinematics. Location 3 was associated with notably lower PLAs and higher PRVs than other locations. This location is furthest offset from the head CG due to the aerodynamic, elongated design of many helmets, suggesting the greater EPS thickness enhances mitigation of normal forces and PLA. This greater distance from the CG also creates a greater moment arm, increasing PRV. Because lower PLAs and higher PRVs offset in the concussion risk function, location 3 produced middle-ground risks compared to other locations. Conversely, although location 2 did not average the lowest kinematics overall, the combination of consistently low PLAs and PRVs at this location produced significantly lower risks. Location 2 falls at the helmet rim, below the testable region in standards. The present results suggest this location offers suitable impact protection nonetheless. However, previous work has shown that impacting this region at higher normal velocities (including those in standards) may cause some helmets to bottom out and produce extreme risk of injury.^3^

The wide-ranging kinematics and concussion risks generated STAR values spanning from 10.9 to 25.3. A helmet’s STAR value estimates the number of concussions that might occur out of all simulated impacts by combining the individual concussion risk from each impact with the relative real-world exposure of that impact. Per 100 impacts, the low and high velocities encompassed 38.0 and 9.4 impacts, respectively (47.4 overall). Although the lower concussion risks associated with the low-velocity impacts indicate that people are less likely to be concussed at this impact severity, the increased frequency at which the overall population experiences these impacts reflects that the total number of concussions occurring at this level could be comparable to the number occurring at the less frequent high-velocity impacts. STAR represents the combined number of concussions, with a possible maximum value of 47.4. The Ballista MIPS thus might reduce the number of concussions to 10.9, while the Watts might reduce the number to 25.3. The lower STAR value reflects lower overall kinematics, with the Ballista MIPS producing considerably lower average PLAs and PRVs than the Watts at both velocities.

**Figure 8:**
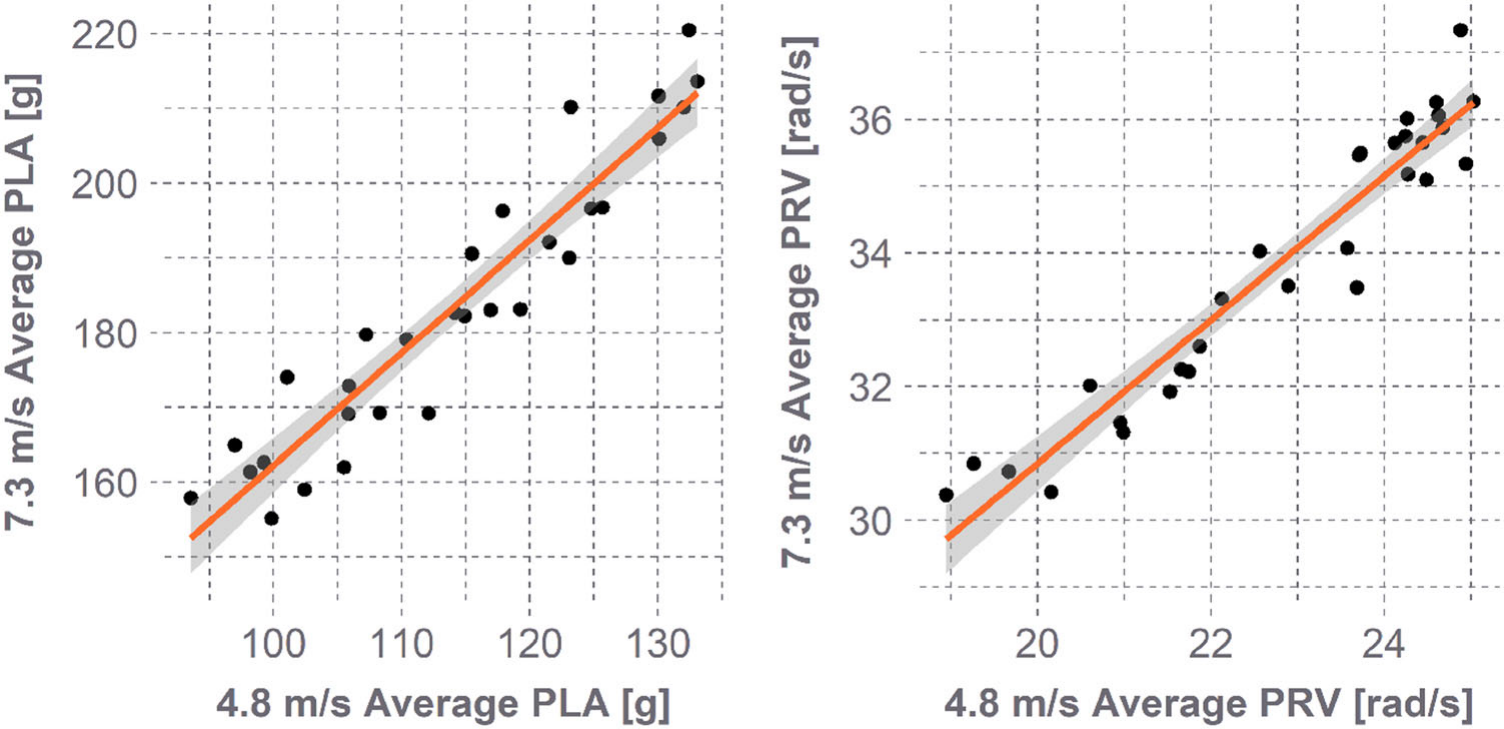
Average PLA (left) and PRV (right) values per helmet at 7.3 m/s vs. 4.8 m/s. Each point represents one helmet’s average values. Shaded regions represent the 95% prediction interval. Strong correlations were observed, suggesting that a helmet’s ability to reduce impact kinematics relative to other helmets is similar at both velocities.

On average, impact velocities and locations contributed relatively equal amounts to a helmet’s STAR value. Location 2 contributed the least to STAR due to lower concussion risks overall. Although both velocities contributed evenly to STAR when averaged across helmets, of particular interest was whether a tradeoff in performance at the two velocities existed for individual helmets. Bicycle helmets are designed to protect against the severe conditions imposed by standards, and it is often questioned whether ensuring that a helmet offers superior protection at more severe energies necessitates offering inferior protection at lower energies, or *vice versa*. To investigate this, each helmet’s average PLA and PRV were determined at both velocities and Spearman correlations between 4.8 and 7.3 m/s results were assessed (Fig. 8). Strong correlations were observed (*ρ *> 0.93, *p *< 0.01), suggesting that helmets that reduced kinematics better at the high velocity also reduced kinematics better at the low velocity, and that no performance tradeoff is evident at the energy levels assessed for these helmet models.

Trends between helmet design and STAR were also assessed. More expensive helmets were associated with lower STAR values, which could be attributed to the higher price of MIPS helmets. However, removing MIPS helmets and repeating the correlation revealed a slightly stronger, equally significant trend (non-MIPS: *R *= − 0.56, *p *= 0.01, all helmets: *R *= − 0.47, *p *= 0.01). This suggests that more expensive helmets offer enhanced protection, although considerable scatter was observed in these data. Style and MIPS also had significant effects on STAR. Style mainly affected STAR via its influence on PLA, with urban helmets producing higher PLAs, particularly at locations 3 and 4. The larger PLAs at these locations may relate to their distance from the helmet rim. For stiffer shells, higher energy is required to buckle the shell and crush the EPS at locations further from the rim or venting.^24^ As most urban helmets contain thicker shells and less venting than road helmets, shell stiffness likely has more pronounced location effects. Conversely, style minimally affected PRV. Interactions between style and MIPS did not have significant effects on PLA or PRV (*p *> 0.13).

The effects of MIPS on STAR were primarily related to differences in PRV. PRV for MIPS helmets was ~ 14% lower at 4.8 m/s and ~ 11% lower at 7.3 m/s. MIPS-location interactions were not significant (*p* = 0.08), nor were MIPS-velocity interactions (*p *= 0.50), suggesting MIPS reduced PRV across all locations and velocities. These results suggest that slip-planes can be an effective technology in helmets by reducing rotational impact kinematics. Another study comparing impact performance across bicycle helmet models using a similar test setup also found MIPS to be effective.^40^ Conversely, an additional study demonstrated higher PRA for MIPS helmets compared to non-MIPS helmets.^1^ The latter study incorporated a HIII neck, which likely influenced results. Further, only two MIPS helmets were included, and both produced greater PLAs, suggesting that additional design factors led to reduced impact protection overall.

All of the helmets in the present evaluation were CPSC-certified. This standard ensures that helmets minimize risk of catastrophic, linear acceleration-related injury under severe normal loading, and requires testing using a variety of environmental conditions and anvil shapes.^9^ The variety in test conditions and focus on severe impacts/injuries make safety standards invaluable in guaranteeing adequate helmet safety. The STAR evaluation can provide supplementary information by indicating relative helmet performance in the most common real-world crash conditions. Real-world conditions are more complex than standards tests, involving oblique loading, rotational head kinematics, and often producing milder injuries such as concussion. Establishing a database of helmet STAR values through a central website or other means provides consumers with relative helmet performance rankings in terms of common impact conditions and concussion risks, while standards certifications ensure helmets meet adequate safety requirements.

There are several limitations associated with the present study. First, although the impact configurations are comprehensive and based in real-world data, a finite number of configurations can be evaluated practically. There likely exist other scenarios in which helmets offer varying levels of protection. Helmets do not reduce kinematics in non-impact scenarios, which can produce rotational-related injuries (including concussion) in extreme cases. Second, the concussion risk function was derived from head impacts to American football players, who experience differing impacts from cyclists (although similar in duration and magnitude) and have skewed physical characteristics compared to the general population.^33^ Nonetheless, a study of concussions in automotive crashes showed the previous PLA and PRA-based version of the risk function to be a better predictor of concussion than other common injury metrics.^21^ The function used herein also relies on a transformation from PRA to PRV based on trends in the underlying dataset.^36^ Although these trends were strong, this transformation may limit the function’s accuracy. A cyclist-specific risk function would provide an optimal basis for evaluating bicycle helmets; however, real-world cyclist head impact data paired with injury outcomes are sparse at present. Nonetheless, the present function is based in one of the largest databases of concussive vs. non-concussive helmeted head impacts. It therefore provides a sound basis for comparing helmet performance, ultimately assigning helmets that produce lower head kinematics a lower risk.

Other limitations pertain to testing boundary conditions. First, although simulating road surfaces using a sandpaper-coated steel anvil represents the industry standard,^11^,^26^,^27^,^40^ this setup is likely overly stiff and may have inflated results.^4^ Second, testing was conducted without an ATD neck, as cyclist oblique impacts may subject the neck to considerable axial compression, a scenario under which the HIII neck is overly stiff.^28^,^38^ These compressive loads would be most severe for impacts toward the top of the helmet. Testing without the HIII neck ensured that results at these locations were not biased by the unrealistic neck response. While some finite element (FE) studies have suggested that activated human neck musculature increases the overall effective mass during a head impact,^12^,^14^ effects were small when considering effect sizes related to injury.^12^ Matched oblique impact tests with and without a HIII neck showed larger influences on head kinematics than a similar FE study comparing human neck effects on cyclist oblique head impact kinematics.^2^,^14^ A previous oblique impact study using the HIII neck required greater normal velocities to produce the same PLAs as the present study.^1^ It should thus be noted that the present kinematics and risks may be larger compared to tests with a neck. As advanced ATD necks are developed with improved biofidelity in axial compression, their effects in oblique impacts should be investigated.

A further limitation is that helmets were tested without extraneous attachments such as visors, which could affect resulting kinematics.^6^ Helmet fit was not directly quantified but rather replicated as a consumer might fit their own helmet. This may have increased variance in the results, although previous work has suggested that fit does not systematically alter cyclist head impact kinematics.^43^ Additionally, categorizing helmets by style and MIPS resulted in small sample sizes for some categories (i.e., only two urban MIPS helmets), limiting the generalizability of results. Some helmets marketed for multi-sport or mountain biking were categorized as road helmets due to similar structural designs; however, these helmets often possess some design distinctions (e.g., increased coverage and stiffer visors for mountain biking helmets) that may affect impact attenuation capabilities. As consumers often choose helmets based on intended activities, these effects should be investigated as more helmets are evaluated using the STAR protocol.

Presently, consumers have limited access to objective biomechanical data differentiating bicycle helmet impact performance. As bicycle helmets are a safety feature, such information should be made available to facilitate educated purchasing decisions. The bicycle STAR evaluation system provides a basis for disseminating helmet performance data that is founded in common cyclist head impact conditions and relevant concussive injury mechanisms. A large range in STAR values across 30 popular helmets on the US market was found, enabling assessment of the influence of design features on a helmet’s ability to reduce concussion risk. Evaluation protocols such as these can supplement standards, which ensure helmets reduce injury risk in severe accident scenarios, by indicating helmet protective capabilities in the most common impact conditions as well. This serves as a challenge to manufacturers to develop innovative helmet designs that push the boundaries of improved protective capabilities. In this way, the bicycle STAR evaluation system has the potential to provide knowledge to consumers regarding helmet safety and can continually encourage the design of safer helmets.
